# Developing and validating an implantable suture tension sensor

**DOI:** 10.1016/j.heliyon.2024.e28907

**Published:** 2024-04-03

**Authors:** F.P.J. den Hartog, Y. Yurtkap, J. Vlot, J.F. Lange, P.J. Tanis, G.J. Kleinrensink

**Affiliations:** a Department of Surgery, ErasmusMC, University Medical Center, Rotterdam, the Netherlands; b Department of Pediatric Surgery, ErasmusMC, University Medical Center, Rotterdam, the Netherlands; c Department of Surgery, Amsterdam UMC, University of Amsterdam, Amsterdam, the Netherlands; d Department of Neuroscience, ErasmusMC, University Medical Center, Rotterdam, the Netherlands

**Keywords:** Suture tension, Biomedical sensor, Incisional hernia, Laparotomy

## Abstract

**Introduction:**

Suture tension has a direct influence on the sutured tissue. For abdominal wall closure, suture tension should be optimal without causing tissue necrosis, which can result in surgical site infection or incisional hernia. The purpose of the present study is to evaluate a device that can measure suture tension in-situ and in real-time.

**Materials and methods:**

A cheap, commercially available analog-to-digital converter was used, in conjunction with a force sensing resistor. A sensor probe housing was designed and 3D-printed. In order to test the sensor, a mechanical, computer controlled human abdominal wall model called the AbdoMAN was used.

**Results:**

An implantable suture tension sensor was developed, keeping cost-effectiveness in mind. This sensor can translate tension in the suture into a downward force, applied to the force sensing resistor. The sensor's raw readout was characterized using a set of weights, from which a formula correlating the readout to a specific force, was derived. Preliminary validation was successfully performed using the AbdoMANmodel, which showed a progressive rise in suture tension when the intra-abdominal pressure was artificially increased over time.

**Conclusion:**

The implantable suture tension sensor appeared to be capable of recording real time changes in suture tension, and the. validation process of this sensor has been initiated. With the information from devices like this, a much better understanding of the issues at play in the development of incisional hernia can be gained.

## 1 Introduction

A midline incision is the most frequently used incision in order to achieve exposure of the organs in the abdominal cavity. Although surgeons always strive for optimal closure, we still see between 5 and 20% incisional hernias (IH) and 17% surgical site infections (SSI). [1,2]. An estimated 300,000 incisional hernia repairs are performed each year in the USA. [3]. IH and SSI can lead to increased morbidity and mortality, leading to $3 billion in increased medical costs in the USA alone [3]. Both IH and SSI are supposedly linked to suturing technique. [1]. Even the most experienced surgeons are not able to perfectly replicate the exact same suture tension every time. [4,5].

Currently, it is suggested that closure of a midline incision is best performed with the small bites technique. [6]. Additionally, the use of a prophylactic mesh for the closure of a midline laparotomy in high-risk patients is suggested. [7,8]. However, there is currently no clear consensus on the best suturing technique. Reported variables related to suturing technique are: bite size, distance between sutures, suture length to wound length ratio, suture materials and usage of interrupted or continuous sutures. [1,6,[8], [9], [10]]. These variables all contribute to the net tension on the sutures used to close an incision, which in turn influences the chance of an incisional hernia. Proper incisional closure involves bringing the two wound edges together, in order for tissue healing to occur. If, however, the sutures are too loose, the risk of wound dehiscence increases. Another reason for the loosening of sutures could be the phenomenon of material creep, in which the suture is plastically deformed due to increased tension over time. [11]. A cause of increased intra-abdominal pressure (IAP) and subsequently increased suture tension over time could be sitting up, coughing or ileus leading to postoperative abdominal distension. [12,13]. Inadequate closure is also seen when sutures are too tight. In this case, the suture can actually cut through healthy tissue and can then cause more inflammation and possibly introduce necrosis to an already weakened area, resulting in worse tissue healing and an increased risk of infection. [5,8].

The aim of this study is to develop an implantable suture tension sensor. Measuring suture tension during incision closure would allow us to decrease the occurrence of the aforementioned complications and to improve overall wound healing. Monitoring suture tension for a longer period of time (hours to days) postoperatively could help detect poorly healing wounds in a minimally invasive way, before macroscopic changes to the tissue are able to indicate this. Furthermore this device could play an important role in education and training settings. Visual feedback on suture tension would be important for interns and residents to quickly and efficiently learn safe closure of surgical wounds.

## 2 Materials and methods

### 2.1 Sensor probe

At the core of the sensor probe lies an Interlink Electronics Force Sensing Resistor (FSR) 400 (product number 30–49649, with solder tabs) Series Force Sensing Resistor. It has an actuation force of approximately 0.2 N–20 N, which is a wide enough range for measuring tension in sutures. [11]. The FSR measures the applied force to a circular area by acting as a variable resistor in an electric circuit. When no force is applied, the resistance across the FSR will be > 10 million Ohms. As the suture tension increases, the force applied to the sensor increases and the electrical resistance will fall exponentially into the thousands of Ohms.

In order to be able to connect a cable to the FSR, an Amphenol ICC FFC**/**FPC (product number 65801-002LF) connector was used. Using this connector allows for easy detachment of the sensor probe from its connector cable. Connector cables were custom-made, using TE Connectivity 20 AWG copper wire (product numbers 400R0111-20-0 and 400R0111-20-9), with Johnson/Cinch Connectivity Solutions (product numbers 108-0902-001 and 108-0303-001) jack plug connectors.

A 3D-model of the case was created using Autodesk Fusion 360, which is 3D-modeling software freely available for educational purposes. [14]. Models were exported as stereolithographic (STL) files and sent to a 3D printer. The 3D printer used to print the models as a Stratasys Objet30 Pro. [15]. The material used was VeroWhite. [16].

### 2.2 Arduino controller

In order to read the raw output from the FSR, an analog-to-digital converter (ADC) was required. The ADC reads the voltage across the FSR, which can be used to calculate the force applied to the FSR. Force was measured in Newtons. The Arduino Uno controller was used, which has a 10-bit ADC, is easily programmable, and has a Universal Serial Bus (USB 2.0A) connector for power supply and serial communication. [17]. It is possible to connect up to 6 tension sensors to one Arduino Uno board. TinkerCAD, a free online computer-aided design (CAD) program, was used to create schematic representations of the circuit. [18].

### 2.3 Measuring model

Suture tension was measured in a mechanical, computer driven/controlled model of the human abdominal wall, named the AbdoMAN. [13]. The AbdoMAN features a specifically designed synthetic material, which mimics the characteristics of a human abdominal wall. It can be insufflated to simulate an increase in IAP. Additionally, it has lateral so called “air muscles”, capable of producing up to 660 N of force, increasing the IAP by up to 74.9 mmHg. [13]. Underneath the AbdoMAN synthetic abdominal wall, a 3500 mL Vacufix collecting bag was placed. During experiments, this bag was insufflated with an Olympus UHI-3 High Flow Insufflator up to a maximum of 20 mmHg.

### 2.4 Data collection

The Arduino controller needed to be programmed to send data from the tension sensor via the serial interface (USB cable). These programs were created in the C programming language, using the Arduino IDE (version 1.8.5). Appendix A contains the program specifically designed for the tension sensor. Data sent by the Arduino controller via the serial interface is then received and processed by a software suite which was custom-made for the tension sensor (see Appendix D). The Python programming language was used to create this software suite. [19]. Pip, a Python package repository, was used to download additional Python packages pySerial and matplotlib. [[20], [21], [22]]. Python packages are bundles of Python code which extend the functionality of the Python programming language. PySerial is a package which was used to receive tension sensor data via the serial interface. Matplotlib is a package which can be used to create and heavily customize interactive graphs. In this case, it was used to create a live, scrolling graph of the tension sensor data. [22].

### 2.5 Data processing

Raw data from the FSR consisted of the activation level of the connected sensor(s) and the current timestamp. It was recorded with a frequency of 50 Hz. Because the Arduino has a 10-bit ADC, a value between 0 and 1023 was given. Thus, the sensor has a resolution of 2^10^ = 1024 different values. The source voltage (V_source_: supplied via USB-cable, connected to a laptop) was determined as 5.06 V, by using a multimeter.

The raw value has a linear relationship to the voltage across the FSR, but an inversely exponential relationship to the force. In order to determine the force (Newton), several steps have to be taken.

Firstly, the raw value (n) needs to be mapped to a voltage, using the following formula:

$$V_{FSR}=n*\frac{V_{source}}{1023}$$

(V_FSR_: FSR voltage, n: sensor activation level, V_source_: Arduino power source voltage)

Subsequently, the resistance across the FSR needs to be calculated, making use of the previously calculated voltage (V_FSR_). In order to do this, the following formula is used:

$$R_{FSR}=R_{pulldown}*\left(\frac{V_{source}}{V_{FSR}}\right)$$

(R_FSR_: FSR resistance, V_FSR_: FSR voltage, R_pulldown_: pulldown resistor, see ***(D)*** in the circuit diagram in appendix C, V_source_: Arduino power source voltage)

The final step is to associate a certain R_FSR_ with the amount of force in Newton applied to the FSR. This calibration was done by suspending known amounts of weights on a piece of string, across the FSR.

## 3 Results

### 3.1 Sensor

**Fig. 1:**
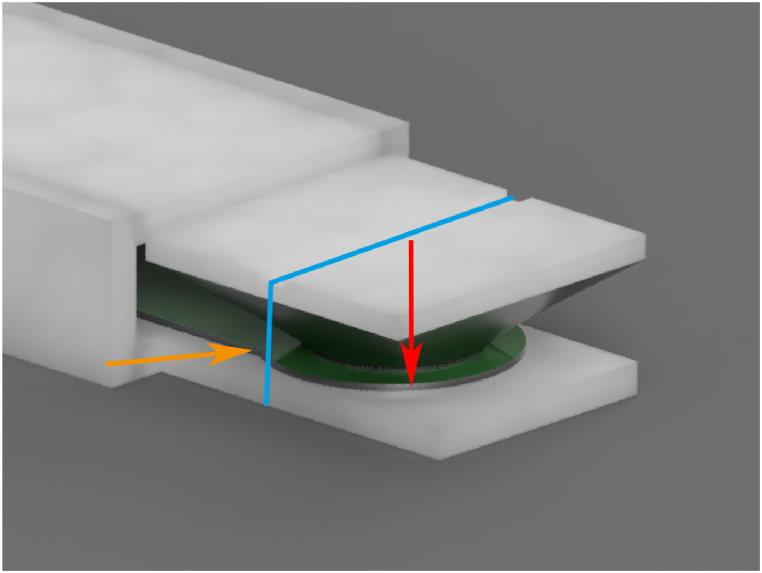
3D-model of the sensor actuator. The tension in the suture (suture: blue; tension direction: orange arrows) is translated to a downward force, applied to the FSR (red arrow).

A schematic overview of the complete sensor system is visible in appendix C. The final implantable sensor probe measures 45 x 12 × 5 mm. In order to measure the tension in the suture with the tension sensor, a physical translation of force was required. By attaching an actuator between the suture and the circular area of the FSR, an increased tension in the suture would translate to a higher measured force by the FSR. The actuator features a notch in the exact center line, allowing the suture to sit tightly and securely in the sensor case. Fig. 1 and appendix B show this mechanism. The tension generated by the suture that lies in the actuator notch is translated downward, onto a circular surface, precisely and evenly distributed, pressing down on the force-sensing part of the FSR.

### 3.2 Experimental setup

**Fig. 2:**
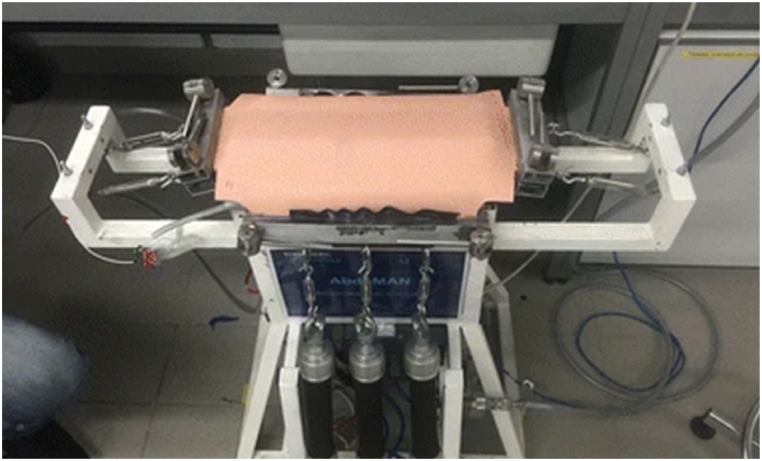
The tension sensor in the experimental setup: the AbdoMAN.

**Fig. 3:**
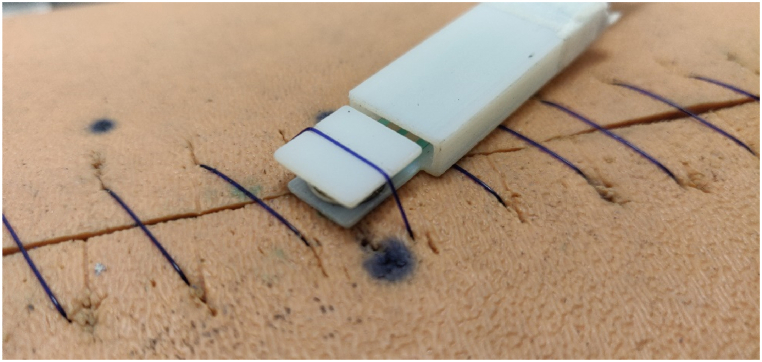
PDS-II (polydioxanone, absorbable, monofilament) 2-0 sutures are visible below the sensor, as well as across the actuator notch. The tension in this suture was being measured.

In order to test the applicability of the tension sensor, an experimental setup mimicking a real, dynamic human abdominal wall was used. An incision representing a midline laparotomy was already present in the synthetic AbdoMAN abdominal wall, across which four evenly spaced interrupted sutures were applied. A fifth suture was placed precisely halfway along the length of the wound and the tension sensor was connected to this suture (see Fig. 2, Fig. 3). Two types of experiments were designed.

(1) a short experiment, which was dynamic in terms of IAP variation and
(2) a long experiment, with a static IAP.

During these experiments, the suture tension was constantly measured and timestamps were marked digitally when the IAP would change. In this way, it was possible to correlate between variations in IAP and suture tension changes. The first step in both experiments was to (re-)apply sutures in the manner described above.

Regarding experiment type 1, for this short, dynamic experiment, the measurement started at 0 mmHg, for 2 min. After these initial 2 min, the IAP was raised by 5 mmHg steps, every 2 min. At 20 mmHg, the IAP was lowered down to 0 mmHg and another 2 min were measured. Appendices E and F show an example of raw, unprocessed measurements for this experiment. Regarding experiment type 2, this long, static experiment also started at 0 mmHg for 2 min. However, after this initial period, the IAP was raised directly to 20 mmHg, where it remained for 30 min. Appendix E shows a representative example of measurements for this experiment. At the marked intervals, where the IAP rises and falls, we could see the measured suture tension rise and fall with it.

#### 3.2.1 Sensor calibration

In order to convert from the raw values gathered in the experiments to force, each tension sensor needed to be calibrated once before use. When the sensor's characteristics are known, we can use these to convert the sensor output into an actual force in Newton. Known forces were applied to the sensor via a series of weights, and the average observed raw value over 60 s of measurement is noted. Doing this with 10g, 20g, 50g, 100g, 200g, 300g, 400g, 500g, 600g, 700g, 800g, 900g and 1000g allowed us to accurately model the sensor's behavior. A formula was extracted from this model, which was used to convert between sensor output and force for this particular sensor unit. When a new sensor is constructed, it will need to be calibrated and it will have its own, different characteristics. As mentioned before, one controller can support up to 6 different tension sensors, so it is possible to measure up to 6 different sutures with one controller.

Sensor drift was considered, especially when a single sensor was used repeatedly in experiments, comprising many hours of usage under tension. According to the sensor's datasheet, the reported sensor drift is <5% per log_10_(time) when placed under 1 kg of load for a period of 35 days. There were no specific drift thresholds set for our experiments, but when a sensor displays values that were inconsistent with previous measurements, this could be attributed to drift. No drift was observed when the sensor was not under any load. Because of the low cost (less than €10,- each) of the FSR 400 sensor, the easiest solution to counter sensor drift is to replace the sensor with a new, calibrated sensor.

## 4 Discussion

### 4.1 Characteristics and qualities of the experimental sensor

A simple, cost-effective and easy to use solution for measuring suture tension was developed. It is now possible to use the sensor to start collecting large amounts of experimental data, using different types of suture material and suturing techniques. This will allow us to objectively measure the different qualities of the suture material and to compare new experimental types of suture to the current standard modalities. Due to the relatively short construction time and low cost, it is possible to produce an amount of tension sensors as large as required.

### 4.2 Cost-effectiveness

The parts used in creating the tension sensor were relatively inexpensive. An estimation of the total cost of the controller, combined with a single sensor probe lies below €150. Considering the enormous costs associated with incisional hernia, finding the optimal closure technique with such inexpensive devices would likely be cost-effective. [3].

### 4.3 Limitations

After each consecutive experiment, sutures were re-applied. This was required in order to guarantee optimal and consistent suture condition before every experiment. However, as we know that even the most experienced surgeon currently does not place every suture in the exact same manner, this does introduce variation in the suture tension before each experiment. [4,5]. This can be circumvented by looking at relative changes in suture tension, and not absolute changes. Experiments so far have all taken place under completely “dry”, i.e. *in vitro* circumstances. One could imagine that experimenting in biological tissue could bring parts of the sensor probe in contact with moisture. It is still unknown to how much moisture and for which amount of time the device can be exposed to, before it stops functioning. If the sensor case performs inadequately under these circumstances, the sensor case design should be adapted, or waterproofing measures such as a silicone sheath need to be applied.

Another factor to consider when measuring different tissues is the stiffness or compliance of the sutured tissue. With increasing IAP, highly distensible tissue will increase suture tension less steeply compared to very stiff tissue.The maximum tension however should theoretically not be different with different tissues at the same IAP as wall-stress of the compartment is the same. Using the relative tension (compared to a baseline tension), rather than the absolute suture tension may perhaps be a more useful parameter. Nonetheless, comparing measurements between two different tissues should always be done with caution, until we have more experience using the sensor in different settings.

### 4.4 Future prospects

The current experimental setup works and repeated measurements have shown to be reliable. It can be used to compare many different types of tissue, suture, suturing techniques, suture patterns or other incision closure techniques like mesh-augmented repair. Data gathered from these experiments can be used to gain insight into and reach a consensus about the intrinsic quality of incision closure techniques. Ultimately, determining the superior abdominal wall closure technique objectively, through experimental studies with the suture tension sensor, can help decrease the incidence of incisional hernia after laparotomies. Aside from the financial burden caused by incisional hernia, patient burden cannot be understated. The sensor could be used to rapidly test a series of different techniques in controlled, pre-clinical experiments, before advancing to prospective studies or clinical trials, investigating the techniques that performed best in the experimental setting.

A different future step in the development of the tension sensor would be to design an experiment on long-term wound healing. Not IAP variations, but actual tissue changes might be assessed to determine the change in suture tension. [23]. The tension sensor can then be applied as a tool to monitor wound healing, thereby probably highlighting or predicting critical moments in wound healing, which would otherwise pass unnoticed or would only be noticed as clinical signs of IH.

Although the current sensor probe is already quite small in size, it is possible to further decrease its size. Shorter variations of the Interlink Electronics FSR400 exist and are currently being tested for implementation into the tension sensor device. This could shorten the total length of the device by tens of millimeters. This does require a redesign of the case and a new series of applicability experiments.

## 5 Conclusion

An implantable suture tension sensor was successfully created. It is capable of measuring both subtle and large variations in suture tension, caused by controlled variations in IAP in a model of the human abdominal wall. By calibrating the sensor, accurate measurements of suture tension are possible. With the information from devices like this, a much better understanding of the issues at play in the development of incisional hernia can be gained.

## Source(s) of funding

None.

## Data availability statement

Data used in this study are available upon reasonable request to the corresponding author.

## CRediT authorship contribution statement

**F.P.J. den Hartog:** Conceptualization, Data curation, Investigation, Methodology, Writing – original draft, Writing – review & editing. **Y. Yurtkap:** Conceptualization, Data curation, Formal analysis, Writing – original draft, Writing – review & editing. **J. Vlot:** Conceptualization, Software, Supervision, Writing – review & editing. **J.F. Lange:** Conceptualization, Investigation, Methodology, Writing – review & editing. **P.J. Tanis:** Supervision, Writing – review & editing. **G.J. Kleinrensink:** Conceptualization, Resources, Supervision, Visualization, Writing – original draft, Writing – review & editing.

## Declaration of competing interest

The authors declare that they have no known competing financial interests or personal relationships that could have appeared to influence the work reported in this paper.
